# Isolation of polysaccharides from *Dendrobium officinale* leaves and anti-inflammatory activity in LPS-stimulated THP-1 cells

**DOI:** 10.1186/s13065-018-0480-8

**Published:** 2018-10-31

**Authors:** Min Zhang, Junwen Wu, Juanjuan Han, Hongmei Shu, Kehai Liu

**Affiliations:** 1 Department of Biopharmaceutics, College of Food Science and Technology, Shanghai Ocean University, 999 Hucheng Ring Road, Lingang New City, Shanghai, 201306 China; 2 National Experimental Teaching Demonstration Center for Food Science and Engineering, Shanghai Ocean University, Shanghai, 201306 China

**Keywords:** *Dendrobium officinale*, Polysaccharides, THP-1 cells, Anti-inflammatory properties, LPS/TLR-4 signal pathways

## Abstract

*Dendrobium officinale* stem is rich in polysaccharides, which play a great role in the medicinal effects of this plant. However, little was known about the polysaccharides from *Dendrobium officinale* leaves. Two kinds of polysaccharides in the leaves, DLP-1 and DLP-2, were obtained by hot water extraction, alcohol sedimentation and chromatographic separation (DEAE-52 cellulose column and Sephadex G-100 column). The average molecular weights were determined as 28,342 Da and 41,143 Da, respectively. Monosaccharide compositions were analyzed using gas chromatography–mass spectrometer. DLP-1 was composed of d-(+)-galactose, dl-arabinose, and l-(+)-rhamnose with a molar ratio of 3.21:1.11:0.23, and traces of d-xylose, d-glucose, and d-(+)-mannose. DLP-2 was consisted of d-glucose and d-(+)-galactose with a molar ratio of 3.23:1.02, and traces of d-xylose, dl-arabinose. Then, we established inflammatory cell model by LPS acting THP-1 cells to investigate the anti-inflammatory effects of DLP-1 and DLP-2. The results indicated that DLP-1 (5 μg/mL) and DLP-2 (50 μg/mL) were effective in protecting THP-1 cells from LPS-stimulated cytotoxicity, as well as inhibiting reactive oxygen species formation. In addition, both DLP-1 (5 μg/mL) and DLP-2 (50 μg/mL) significantly suppressed toll-like receptor-4 (TLR-4), myeloid differentiation factor (MyD88) and tumour necrosis factor receptor-associated factor-6 (TRAF-6) mRNA and protein expression in LPS-stimulated THP-1 cells.

## Introduction

*Dendrobium officinale* Kimura et Migo belongs to *Dendrobium* Sw., Orchidaceae and widely distributes in the tropical and subtropical areas [1]. The stem is medical part of *Dendrobium officinale* in China and included in Chinese Pharmacopoeia [2]. Its stem is usually processed into one of the traditional Chinese medicine named “Tiepishihu” after twisted into a spiral while baking and used as a tonic for more than 2000 years due to its exceptional effect [3–6]. Also, it could be either chewed directly or stewed in porridge, soup, and dishes as a high-quality food in diets [7]. However, *Dendrobium officinale* leaves have been used as neither medicine nor food and often discarded as waste, which not only causes environmental pollution, but also wastes this valuable resource.

The current research on *Dendrobium officinale* also focuses on stems, and surprisingly little was known about leaves until now. In fact, the stems and leaves originate from the same plant, so *Dendrobium officinale* leaves should have a great range of potential utilities and a prospect of development in food, medical and health care. For example, *Dendrobium officinale* leaves exhibited good auxiliary therapeutic effect on hypertension, hyperglycemia, hyperlipidemia and other similar symptoms as well as promoting health when serving as tea [8, 9], and there was also a research indicated that *Dendrobium officinale* leaves could enhance the T lymphocyte proliferation, the delayed type hypersensitivity and NK cell function of female rats after two generation reproduction [10]. Therefore, *Dendrobium officinale* leaves are also worth researching.

*Dendrobium officinale* stems contain bioactive phytochemicals, such as polysaccharide, dendrobine, sesquiterpenoids and volatile components, but the predominant one is polysaccharide [11]. Polysaccharides, along with proteins, nucleic acids and lipids, are primary class of biological macromolecules [12], and are very crucial since they have tremendous medicinal values [13]. Tons of studies on polysaccharides from *Dendrobium officinale* stems have achieved great progress. The polysaccharides from *Dendrobium officinale* stems could exert immunoregulatory activity in vitro by means of promoting splenocyte proliferation, enhancing natural killer cell-mediated cytotoxicity and stimulating of cytokine secretion of both splenocytes and macrophages [14]. In consideration of homology relationship between the stems and leaves, polysaccharides should be main active component in *Dendrobium officinale* leaves and rich in content. So the polysaccharides from *Dendrobium officinale* leaves (DLP) were chose to be the research object of this study. On basis of preliminary studies of polysaccharides in stems, the anti-inflammatory activity of polysaccharides in leaves was investigated in this study. To the best of our knowledge, there is no report on the isolation and anti-inflammatory activity of the polysaccharides from *Dendrobium officinale* leaves in the literature.

THP-1, a human leukemia monocytic cell line, has been extensively modeled and used for investigating anti-inflammatory effects of compounds due to its unique characteristics [15]. The cells were usually stimulated with LPS, being in an activation state. Furthermore, LPS and food compounds were often simultaneously applied to THP-1 cells to investigate food compounds for inflammation modulating effects by gene expression response analysis [16]. In this contribution, we established inflammatory cell model using LPS acting THP-1 cells, by means of which to investigate the effects of DLP-1 and DLP-2 on the cell viability, ROS generation, and the TLR-4, MyD88 and TRAF-6 expression in LPS/TLR-4 signal pathways, including mRNA and protein expression, to explore these two polysaccharides’ anti-inflammatory activity and mechanism.

## Results

### Isolation of polysaccharides DLP-1 and DLP-2

**Fig. 1:**
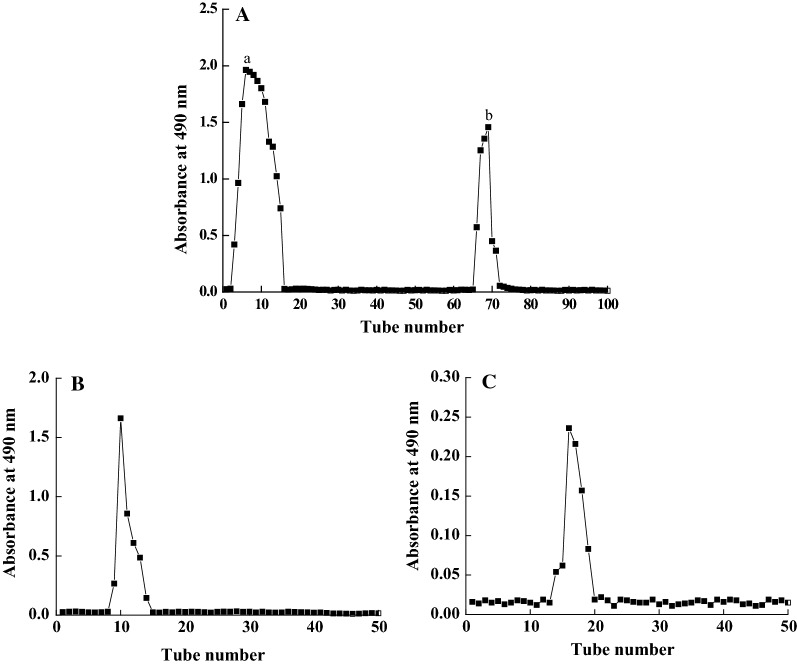
Purification of polysaccharides. **A** DEAE-52 anion-exchange column chromatography elution curve of crude polysaccharide extracted from *Dendrobium officinale* leaves. **B**, **C** Sephadex G-100 column chromatography elution curves of fraction a and b

Two completely separated fractions, a and b, were obtained after DLP was eluted through a DEAE-52 anion-exchange column (Fig. 1A) and further purified by Sephadex G-100 gel filtration column. Their elution curves in Fig. 1B, C were displayed as two single narrow symmetrical peaks, explaining for homogeneous components polysaccharides denominated as DLP-1, and DLP-2.

### Molecular weight and monosaccharide composition of DLP-1 and DLP-2

The average molecular weight and monosaccharide composition were determined by GPC and GC–MS. The standard sample of PEG was used for calibration curve establishment. The results showed that the average molecular weight of DLP-1 and DLP-2 were 28,342 Da and 41,143 Da, respectively (Table 1). DLP-1 was consisted of d-(+)-galactose, dl-arabinose, and l-(+)-rhamnose in a mole ratio of 3.21:1.11:0.23, and traces of d-xylose, d-glucose and d-(+)-mannose. DLP-2 was consisted of d-glucose and d-(+)-galactose in a mole ratio of 3.23:1.02, and traces of d-xylose and dl-arabinose (Table 2).

**Table 1:** GPC analysis of DLP-1 and DLP-2

Dist name	Mn	MW	MP	MZ	MZ + 1
DLP-1	17,382	28,342	8294	44,869	60,951
DLP-2	24,328	41,143	12,613	81,563	134,773

**Table 2:** GC–MS analysis of DLP-1 and DLP-2

Monosaccharide	Fragment area percent (%)		Mole ratio	
	DLP-1	DLP-2	DLP-1	DLP-2
l-(+)-Rhamnose	0.75	–	0.23	–
dl-Arabinose	22.37	1.10	1.11	–
d-Xylose	–	0.90	–	–
d-Glucose	–	76.10	–	3.23
d-(+)-Galactose	77.60	28.00	3.21	1.02

### Effects of DLP-1 and DLP-2 on cell viability and ROS generation in LPS-stimulated THP-1 cells

**Fig. 2:**
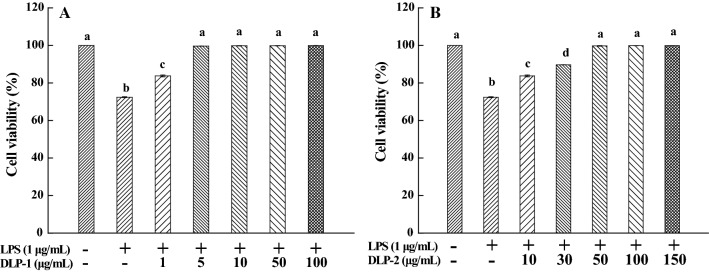
Effects of DLP-1 and DLP-2 on cell viability. **A** Cells were treated with LPS (1 μg/mL) for 24 h in the absence or presence of DLP-1 at different concentrations (1, 5, 10, 50 and 100 μg/mL). **B** Cells were treated with LPS (1 μg/mL) for 24 h in the absence or presence of DLP-2 at different concentrations (10, 30, 50, 100 and 150 μg/mL). Cell viability was measured using MTT assay. Values were mean ± SD (n = 6); bars with the same letter were not significantly different between groups at *P* < 0.05, in accordance with Duncan’s multiple range test

As shown in Fig. 2, LPS-stimulated cytotoxicity could be suppressed by DLP-1 and DLP-2 and this effect appeared to be dose-related. When the concentrations reached 5 μg/mL and 50 μg/mL, DLP-1 and DLP-2 were able to completely protect the THP-1 cells against LPS-stimulated cytotoxicity, respectively. Thus, the concentrations of DLP-1 and DLP-2 were chosen for further research of anti-inflammatory activity.

**Fig. 3:**
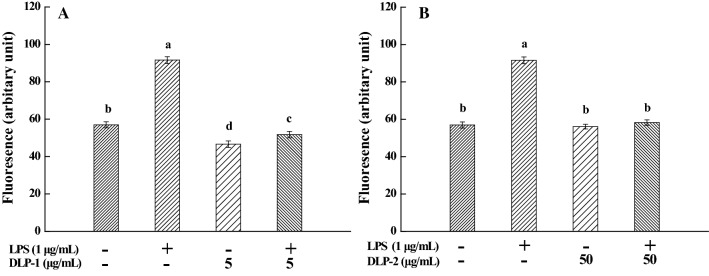
Effects of DLP-1 and DLP-2 on reactive oxygen species (ROS) generation. **A** Cells were treated with LPS (1 μg/mL) for 24 h in the absence or presence of DLP-1 (5 μg/mL). **B** Cells were treated with LPS (1 μg/mL) for 24 h in the absence or presence of DLP-2 (50 μg/mL), followed by addition of 10 μM DCFH-DA to incubate for 30 min. Values were mean ± SD (n = 3), bars with the same letter were not significantly different at *P* < 0.05, in accordance with Duncan’s multiple range test

Compared with untreated THP-1 cells, ROS generation in LPS-stimulated cells increased significantly and the mean fluorescence intensity was enhanced remarkably from 56.98 ± 1.63 (a.u) to 91.59 ± 1.81 (a.u) (Fig. 3). However, the addition of DLP-1 and DLP-2 resulted in a significant reduction of ROS formation in LPS-treated cells (*P* < 0.01). These results indicated that 5 μg/mL DLP-1 and 50 μg/mL DLP-2 could inhibit ROS generation effectively. ROS are known to play an important role in the activation of several pro-inflammatory genes. DLP-1 and DLP-2 exhibited anti-inflammation activity through suppressing LPS-induced ROS generation in this study.

### DLP-1 and DLP-2 influenced the TLR-4, MyD88 and TRAF-6 signal transduction pathways

**Fig. 4:**
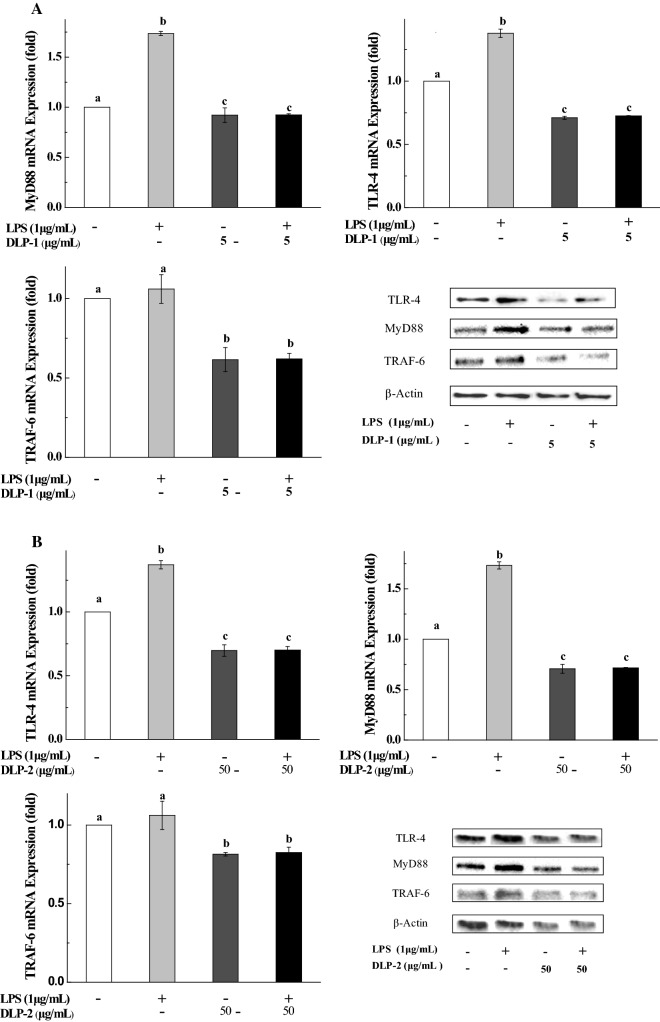
Effects of DLP-1 and DLP-2 on mRNA and protein expression of TLR-4, MyD88 and TRAF-6. Cells were treated with LPS (1 μg/mL) for 24 h in the presence or absence of **A** DLP-1 (5 μg/mL) and **B** DLP-2 (50 μg/mL), respectively. The mRNA expression levels were determined by qRT-PCR. The protein expression was detected by western blotting assay. β-Actin was used as loading control. Values were mean ± SD (n = 3), bars with the same letter were not significantly different at *P* < 0.05, in accordance with Duncan’s multiple range test

From Fig. 4, LPS treatment led to a significant up-regulation of TLR-4, MyD88 and TRAF-6 mRNA expression. When DLP-1 or DLP-2 was added to the LPS-stimulated THP-1 cells, their mRNA expression declined observably, even lower than the original level (the cells treated with nothing).

Consistent with the mRNA expression, marked increase of the TLR-4, MyD88 and TRAF-6 protein expression levels could be observed in the THP-1 cells after treated with LPS alone. After co-treatment with DLP-1 or DLP-2, their protein expression was more or less reduced. These results clearly evidenced that DLP-1 and DLP-2 could inhibit the TLR-4, MyD88 and TRAF-6 at the mRNA and protein levels in LPS-induced THP-1 cells.

## Discussion

It has been known that the polysaccharides from *Dendrobium officinale* stems have good medicinal value [17–19]. Given the homology of the stems and leaves, we initially thought their polysaccharides would have structural similarities, but this was not the case. The preliminary research showed that mannose and glucose were the main monosaccharide components of the polysaccharides in *Dendrobium officinale* stems [3]. In this study, two polysaccharides were isolated from *Dendrobium officinale* leaves. DLP-1 consisted of d-(+)-galactose, dl-arabinose, and l-(+)-rhamnose with a mole ratio: 3.21:1.11:0.23. DLP-2 consisted of d-glucose and d-(+)-galactose with a mole ratio: 3.23:1.02. Obviously, there were significant differences in monosaccharide composition of the polysaccharides between *Dendrobium officinale* leaves and stems.

In addition, inflammation is the body’s self-protective behavior. An appropriate inflammatory response can identify pathogens and be beneficial to the body, but excessive inflammation will do harm to cells and tissues, thereby leading to many diseases, including arthritis, heart disease, cancer, neurological disorders, obesity and diabetes [20]. The polysaccharides extracted from plants represent a structurally diverse class of macromolecules and have been proven to possess a variety of biological activities, especially anti-inflammatory activity [21]. In our research, the polysaccharides from *Dendrobium officinale* leaves were able to completely counteract the effects of LPS-induced cytotoxicity on THP-1 cells, as well as blocking ROS formation, suggesting they can be used as anti-inflammatory agents.

In the study of the anti-inflammatory mechanism, DLP-1 and DLP-2 exhibited anti-inflammatory activities through inhibition of the activation of TLR-4/MyD88/TRAF-6 pathway at mRNA and protein levels. Toll-like receptor-4 (TLR-4) is a type of pattern recognition receptors (PRRs) that recognize conserved pathogen-associated molecular patterns, mainly expressed on cells of the innate immune system. The adapter protein myeloid differentiation factor 88 (MyD88), as an immediate adaptor molecule, plays a critical role in activating IRAK-1 and IRAK-4 of signaling cascades [22, 23]. Upon activation of TLR-4 signaling pathways, MyD88 is recruited by combining with TLR-4, IRAK1, IRAK-4 and forming a complex. Following recruitment to MyD88, the rapid autophosphorylation happens to IRAK-1 and make it dissociate from the signaling complex [24, 25]. Dissociated IRAK1 subsequently interacts with TRAF-6 and triggers the activation of a kinase cascade involving IκB kinase (IKK), which culminates in the phosphorylation and degradation of IκB (NF-κB inhibitor), subsequently empowers NF-κB to enter the nucleus, and triggers pro-inflammatory gene expression [26, 27]. The TLR-4 signaling pathway was considered to play a key role in inflammatory processes [28, 29]. Therefore, therapeutics targeting TLR-4 signaling pathways were expected to safely alleviate chronic inflammatory conditions without compromising the innate immune response [30, 31].

In this study, DLP-1 and DLP-2 significantly suppressed mRNA expression of TLR-4, MyD88 and TRAF-6 in LPS-stimulated THP-1 cells. The western blotting results showed that DLP-1 and DLP-2 down-regulated the protein expression of TLR-4, MyD88 and TRAF-6, suggesting that influences of polysaccharides on protein expression of TLR-4, MyD88 and TRAF-6 were corresponding to the effects on their mRNA expression. In summary, these results demonstrated that DLP-1 and DLP-2 exhibited anti-inflammatory activity by inhibiting the activation of TLR-4/MyD88/TRAF-6 pathway at mRNA and protein levels. This is similar to the polysaccharides in the stems [32]. The biological activities of polysaccharides were intimately relevant to the molecular weight, monosaccharide, glycosidic-linkage composition, functional groups, branching characteristics and conformation [33, 34]. Therefore, the differences in concentrations of anti-inflammatory actions between DLP-1 and DLP-2 might be related to the above factors, which need to be further studied.

## Materials and methods

### Materials and reagents

*Dendrobium officinale* leaves were collected from Zhejiang province of China. RPMI 1640 medium and fetal bovine serum (FBS) were purchased from Invitrogen (Carlsbad, CA, USA). 2′,7′-dichlorofluorescin diacetate (DCFH-DA), lipopolysaccharide (LPS), dimethyl sulfoxide (DMSO), buffered solution (PBS) and 3-(4,5-dimethylthiazol-2-yl)-2,5-diphenyl tetrazolium bromide (MTT) were purchased from Sigma-Aldrich (St. Louis, MO, USA). NaCl, C_2_H_5_OH, NaOH, H_2_SO_4_, HCl, (CH_3_CO)_2_O, TCA, TFA, phenol, hydroxylamine hydrochloride, petroleum ether, pyridine, and chloroform were obtained from Sinopharm Chemical Reagent Co., Ltd (Shanghai, China). TGX Stain-Frree FastCast Acrylamide Kit (TGX FastCast), enhanced chemiluminescence reagents (ECL) and transfer buffer provided by Bio-Rad Laboratories (Shanghai, China). β-Actin, anti-MyD88, anti-TRAF-6 and anti-rabbit IgG antibodies were bought from Cell Signaling Technology (Danvers, MA, USA). Anti-TLR-4 was provided by AbCam (Cambridge, MA, Britain).

### Isolation of polysaccharides

*Dendrobium officinale* leaves were dried and crushed into powder, followed by addition of petroleum ether (1:5, w/v, solid/liquid ratio) to remove fat-soluble ingredients. The skimmed sample was extracted with distilled water (1:30 solid/liquid ratio) at 70 °C for 120 min. Subsequently, aqueous extracts were collected, filtered, evaporated and precipitated by addition of 85% ethanol (4 °C, 24 h). The precipitation was gathered through centrifugation (4 °C, 4000*g*, 20 min), and then freeze-dried. After deproteinized using Sevag’s method, the sample was dialyzed (3500 Da MWCO) and freeze-dried to obtain the crude polysaccharides, named as DLP.

2 mL of DLP solution (100 mg/mL) was added to a DEAE-52 cellulose chromatography column, and then eluted at a 1.0 mL/min flow rate with distilled water and NaCl solution (0.05, 0.1, 0.3, 0.4 mol/L sequentially). 4 mL eluent was collected in each tube. Two completely separated fractions, A and B, were gathered by measuring eluent absorbance at 490 nm according to the phenol–sulfuric acid method. Fraction A and B were dialyzed (3500 Da MWCO) and lyophilized. Then 2 mL of solution A or B (100 mg/mL) was further purified using Sephadex G-100 column. The column was eluted at a 0.5 mL/min flow rate with distilled water and 0.3 mol/L NaCl solution. After dialyzed (3500 Da MWCO) and lyophilized, two purified polysaccharides named DLP-1 and DLP-2 were obtained.

### Determination of molecular weight

The average molecular weights of DLP-1 and DLP-2 were measured by a waters 2695 gel permeation chromatography (GPC) system equipped with three columns of HR3, HR4, HR5 (7.8 × 300 mm) and a Waters 2414 Refractive Index Detector. Sample size was 50 μL, and pure water was used as mobile phase at a flow rate of 1 mL/min. The column temperature was controlled at 40 °C during 45 min of the operation time. The standard curve was established with PEG standard.

### Analysis of monosaccharide composition

GS-MS was used for detecting the monosaccharide composition of DLP-1 and DLP-2. 5 mg of DLP-1 and DLP-2 were accurately weighed,and then mixed with 2 mL TFA (2 mol/L) in sealed ampoule and incubated for 8 h at 120 °C. After the vacuum-rotary evaporation procedure was adopted to remove TFA, the hydrolyzate was dissolved in 0.5 mL pyridine and reacted with 10.0 mg hydroxylamine hydrochloride at 90 °C for 0.5 h. The mixture was cooled to 25 °C and concentrated by rotary evaporator, followed by addition of 1 mL chloroform and centrifugation. 1 μL of supernatant was injected onto GC–MS system (Agilent Technologies, 77890A-5975C, USA) equipped with a DB-5MS column (30 m × 0.25 mm × 0.25 μm, Agilent). The operation parameters were as follows: the N_2_ flow rate 1 mL/min, injection temperature 270 °C, ion source temperature 230 °C. The column temperature was programmed from 100 °C (hold 2 min) to 190 °C at a heating rate of 20 °C/min, and then increased at 3 °C/min to 260 °C, finally at 10 °C/min to 300 °C (hold 4 min). Eight monosaccharide standards (l-(+)-rhamnose, dl-arabinose, l-(−)-fucose, d-xylose, d-allose, d-(+)-mannose, d-glucose and d-(+)-galactose) were handled in the same way.

### Cell culture

THP-1 cells were purchased from Chinese Academy of Science cell bank (Shanghai, China). RPMI-1640 medium containing 10% FBS was used for THP-1 cells culture and the Petri dish were incubated at 37 °C in a 5% CO_2_ atmosphere.

### MTT assay

The effect of DLPs on cell viability was determined by MTT assay. In brief, TPH-1 cells were seeded in a 96-well plate at a density of 1 × 10^5^ cells each well for 12 h, and further treated with DLPs at different concentrations for another 24 h. Then, 180 μL serum-free medium and 20 μL MTT solution (5 mg/mL) were added to the corresponding wells. After 4 h of incubation, 150 μL DMSO was added as a solvent to dissolve formazan crystals. Finally, the absorbance value at 550 nm was quantitated with ELISA plate reader (Model 680; Bio-Rad, Hercules, CA).

### Intracellular ROS assay

The cells were seeded in 24-well plates in RPMI 1640 medium containing 10% FBS and incubated for 18–24 h. Then, the final concentrations of 0.1% DMSO and 1 μg/mL LPS with or without testing sample (5 μg/mL DLP-1 or 50 μg/mL DLP-2) was added to cell wells and incubated for 24 h. Cells were collected by centrifugation at 300*g* for 5 min, washed with PBS twice, and then treated with 10 µM of DCFH-DA in an incubator for 30 min. At last, cells were washed with PBS twice and transferred onto ice for test of flow cytometry.

### Quantitative reverse transcriptase polymerase chain reaction (RT-PCR) analysis

Total RNA was extracted with RNAiso Plus (Takara, Code No. 9108/9109) and the cDNA was synthesized with PrimeScriptTM RT reagent kit with gDNA Eraser (Takara, Code No. RR047A). Then the relative expression content of mRNA was quantified using Applied Biosystems 7500 Real Time PCR System (Applied Biosystems, Foster City, CA, USA). The primers were showed in Table 3. The PCR conditions were as follows: stage 1, 1 reps for 30 s at 95 °C; stage 2, 40 reps for 5 s at 95 °C, for 34 s at 60 °C; stage 3, 1 reps for 15 s at 95 °C, for 60 s at 60 °C, for 15 s at 95 °C.

**Table 3:** PCR primer sequences used in this study

Gene	Sense primer	Antisense primer
GAPDH	5′-AAA TCC CAT CAC CAT CTT CC-3′	5′-GCA GAG ATG ATG ACC CTT T-3′
TLR-4	5′-ATG CCT GTG CTG AGT TT-3′	5′-CTC TAC CAT ACT TTA TGC AGC C-3′
MyD88	5′-CTA GGT GGG AAA GTC CCA TCA-3′	5′-TCT TCC TCT CTC TGT GCT TCA TTA-3′
TRAF-6	5′-TCC TTG CCC TGT TCT CAA T-3′	5′-GCA TGG AAC GTG TGG AT-3′

### Western immunoblot analysis

The treated THP-1 cells were violently shaken for 10 min on ice in 200 µL RIPA Lysis Buffer. BCA Protein Assay Kit was used to quantify the protein contents. The equal amounts of protein samples, mixed with loading buffer and denatured in boiling water, were separated on a 15% SDS-PAGE and transferred to the PVDF membranes. Immune complexes were formed by incubation of the proteins with anti-TLR-4, anti-MyD88 and anti-TRAF-6 primary antibodies overnight at 4 °C. Afterwards, the membranes were rinsed and probed with secondary antibodies. Immunoreactive protein blots were visualized with ECL immunoblotting detection reagents and the bands were then analyzed by ChemiDoc^®^ MP Image Lab.

### Data statistics

Quantitative data were expressed as mean ± standard deviation (SD) from three repeated experiments conducted in a parallel manner. Values were calculated by SPSS Statistics 17.0 in accordance with one way analysis of variance (ANOVA) and Duncan’s multiple range tests. *P* < 0.05 was accepted to be significantly different.
